# *Enterocytozoon bieneusi* detected by molecular methods in raw sewage and treated effluent from a combined system in Brazil

**DOI:** 10.1590/0074-02760160435

**Published:** 2017-06

**Authors:** Sandra Yamashiro, Vagner Ricardo da Silva Fiuza, Ângela Therezinha Lauand Sampaio Teixeira, Nilson Branco, Carlos Emílio Levy, Isabel Cristina Vidal Siqueira de Castro, Regina Maura Bueno Franco

**Affiliations:** 1 Universidade Estadual de Campinas, Instituto de Biologia, Departamento de Biologia Animal, Laboratório de Protozoologia, Campinas, SP, Brasil; 2 Universidade Estadual de Campinas, Faculdade de Engenharia Civil, Arquitetura e Urbanismo, Departamento de Saneamento e Ambiente, Campinas, SP, Brasil; 3 Universidade Estadual de Campinas, Faculdade de Ciências Médicas, Departamento de Patologia Clínica, Hospital de Clínicas, Campinas, SP, Brasil

**Keywords:** sewage, Enterocytozoon bieneusi, PCR, treated effluent, combined system, Brazil

## Abstract

**BACKGROUND:**

*Enterocytozoon bieneusi* are the most common microsporidia associated with different clinical manifestations such as diarrhoea, respiratory tract inflammation and acalculous cholecystitis, especially in immunocompromised patients. Infection usually occurs by ingestion of food and water contaminated with spores, but can also result from direct contact with spores through broken skin, eye lesions, and sexual transmission, depending on the microsporidian species. Although there are reports of *E. bieneusi* found in humans and animals in Brazil, there are no published studies of environmental samples examined by molecular methods.

**OBJECTIVES:**

The purpose of this study was to verify the presence of *E. bieneusi* in raw sewage and treated effluent from a combined system by molecular methods.

**METHODS:**

Raw sewage and treated effluent samples collected from a combined system were analysed for the presence of *E. bieneusi* using the internal transcriber spacer (ITS) region of *E. bieneusi* by nested polymerase chain reaction.

**FINDINGS:**

The analysis revealed *E. bieneusi* presence and a novel genotype (EbRB) in one raw sewage sample and one treated effluent.

**MAIN CONCLUSIONS:**

The presence of *E. bieneusi* in final effluent indicates that the combined system may not remove microsporidian spores. This study is the first report of *E. bieneusi* in environmental samples in Brazil.

Microsporidia represent a group of obligate intracellular parasites nested within the kingdom Fungi, with 1300 to 1500 species in 187 genera (Vávra & Lukes 2013). These ubiquitous organisms have been found in a wide range of vertebrate and invertebrate hosts with at least 17 species reported in humans, in which they can cause the opportunistic disease microsporidiosis (Fayer & Santín-Duran 2014). Faecal-oral transmission by ingestion of food and water contaminated with spores is the most likely route of transmission to humans (Didier & Weiss 2011), whose infections are of commercial, ecological, and medical significance (Weiss & Becnel 2014). Spores are resistant to the environment and may exhibit long survival times (Li et al. 2003).

Microsporidiosis has been reported in all continents except Antarctica (Weiss 2014), primarily in severely immunocompromised individuals (e.g., HIV/AIDS and transplant patients) (Galván et al. 2011). Diarrhoeal episodes in patients with AIDS can range from 30% to 70% (Field & Milner Jr 2015). However, cases in HIV-negative people, tourists, and the elderly are increasing (Galván et al. 2011).

*Enterocytozoon bieneusi* are the most common microsporidia associated with different clinical manifestation such as diarrhoea, respiratory tracts inflammation and acalculous cholecystitis (Didier & Weiss 2006). Spores have been detected in various environmental samples, including surface water (Sparfel et al. 1997, Dowd et al. 1998), swimming pools (Fournier et al. 2002), wastewater samples (Ayed et al. 2012, Ma et al. 2016), and crop irrigation water (Cotte et al. 1999). Moreover, a foodborne outbreak of gastrointestinal illness was caused by food ingestion in over 100 people was associated with the consumption of *E. bieneusi*-contaminated vegetables in Sweden (Decraene et al. 2012).

The epidemiology of microsporidiosis in humans in developing countries is poorly understood (Henriques-Gil et al. 2010, Li et al. 2012); hence there is a great need for clinical and environmental research to better understand *E. bieneusi* distribution and its risks to human health. In Brazil, nine studies have used molecular methods to detect this pathogen in HIV-infected patients presenting chronic diarrhoea, and animals including cattle, pigs, chickens, coatis, pigeons, and exotic birds, which highlights the potential risk of transmission to healthy humans (Brasil et al. 1998, 2000, Feng et al. 2011, Lallo et al. 2012a, b, Fiuza et al. 2015, 2016a, b, Cunha et al. 2016). However, no information regarding *E. bieneusi* presence in environmental samples in Brazil is available.

During January 2013 to June 2014, Costa et al. (2013) verified several cases of microsporidiosis among transplant patients and HIV+ individuals attended by the University Clinical Hospital (University of Campinas, Campinas, Brazil) by microscopy techniques.

In this period, raw sewage and treated effluent samples from a combined system that receives and treats the hospital effluent were preserved to verify the presence of *E. bieneusi*, since diarrhoea was the most prominent clinical manifestation in these patients. Furthermore, *E. bieneusi* is the microsporidian species most commonly found in humans. Molecular analysis was performed and revealed the presence of *E*. *bieneusi* DNA. In addition, a novel *E. bieneusi* genotype (EbRB) was found in one raw sewage sample and one treated effluent sample. Our findings are of great importance to public health, as this is the first report of molecular methods used to determine the presence of *E. bieneusi* in environmental samples in Brazil.

## MATERIALS AND METHODS

*Wastewater samples* - Wastewater sampling was performed from January, 2013, to June, 2014, every two weeks, regarding the hydraulic retention time (HRT) of each reactor. Samples from raw sewage (n = 18) and treated effluent (n = 18) were collected in plastic bottles. The detection of *E. bieneusi* was conducted at the Laboratory of Protozoology, Institute of Biology, University of Campinas.

The raw sewage samples were subjected to a centrifuge-concentration technique described by Cantusio et al. (2006) with minor modifications, including centrifugation at 1500 x *g* for 15 min, and using 1% Tween 80 for homogenisation of samples. Treated effluent samples were subjected to the membrane filtration technique of Franco et al. (2001), using 1% Tween 80 to elute membrane surfaces and a double centrifugation step at 1500 x *g* for 15 min.

*Region studied* - The combined system was operated at a pilot scale in the Biological Processes Laboratory (LABPBIO), located at the University of Campinas, Campinas, Southeast Brazil.

This system was filled with raw sewage that received a discharge of effluent that drains a region of the University of Campinas campus comprising a metropolitan reference hospital, a nursery, financial banks, several cafeterias, and three university restaurants. Over ten thousand people circulate through this area daily.

**Fig. 1:**
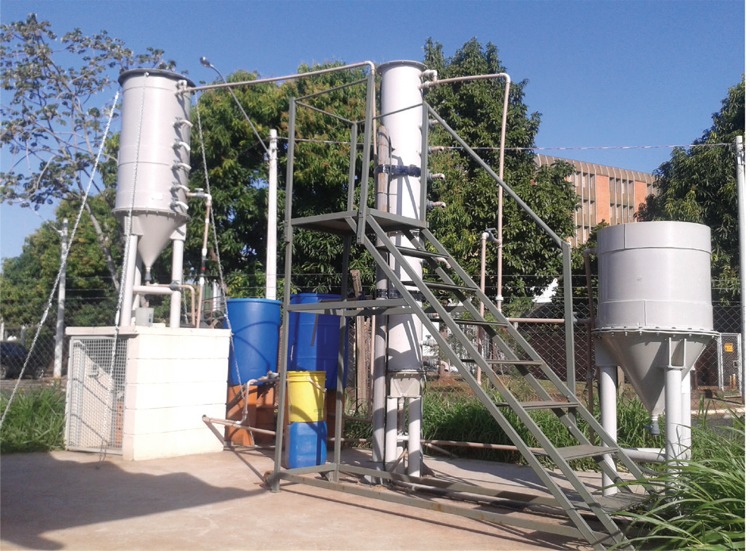
: pilot combined system of wastewater treatment: sewage reservoir (blue tank); from left to right: anaerobic/anoxic filter (AAF) followed by aerated submerged biofilter (ASB) and decanter (DEC) located at the Biological Process Laboratory in University of Campinas (Campinas, São Paulo, Brazil).

**Fig. 2:**
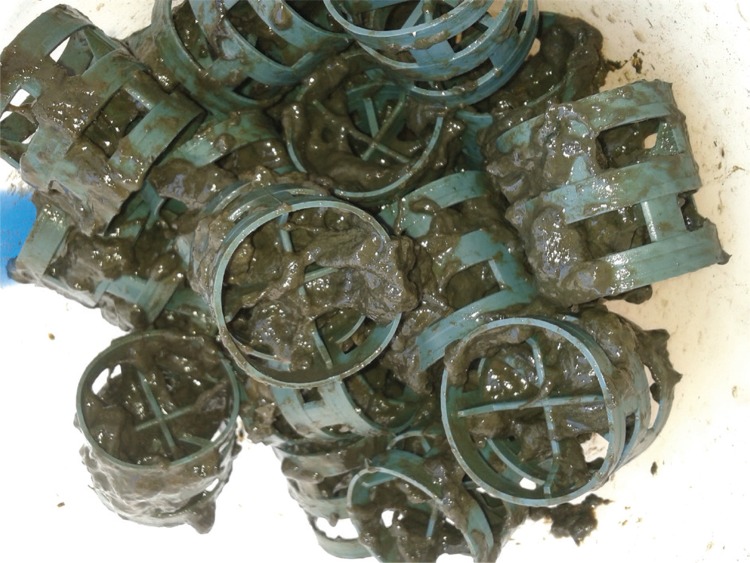
: “Pall rings” in the aerated submerged biofilter.

*Combined system* - The combined system consisted of one trickling filter composed of the following units: an anaerobic/anoxic filter (AAF) with a volume of 298.7 L, an aerated submerged biofilter (ASB) with a volume of 131.5 L, and a decanter (DEC) with a volume of 188.5 L (Fig. 1). Both reactors (AAF and ASB) were fulfilled with “pall ring” type rings (Fig. 2) produced in recycled polypropylene with a dimension of 38 mm x 38 mm (diameter x height), and a surface area of 128 m^2^.m^-3^, and a “free space index” of 89%.

**Fig. 3:**
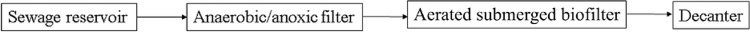
: flow direction of the sewage in the combined system for wastewater treatment.

A volume of 700 L of raw sewage is treated daily by the combined system with a flow rate of 25 L/h (Fig. 3).

*DNA extraction* - DNA extraction of microsporidian spores was performed using the ZR Fungal/Bacterial DNA^®^ kit (Zymo Research, Irvine, CA, USA) according to the manufacturer’s instructions. After extraction, 100 µL DNA was stored at -20ºC.

*Nested polymerase chain reaction (PCR) and purification* - A nested PCR protocol (Buckholt et al. 2002) was used to amplify two fragments (435 and 390 bp) of the internal transcriber spacer (ITS) region of the *E. bieneusi* rRNA gene, with the following primer sets: EB-ITS3: (5’- GGT CAT AGG GAT GAA GAG -3’) and EB-ITS4: (5’- TTC GAG TTC TTT CGC GCT C -3’), for the first PCR; EB-ITS1: (5’- GCT CTG AAT ATC TAT GGC T -3’) and EB-ITS2.4: (5’- ATC GCC GAC GGA TCC AAG TG -3’) for the second PCR.

The PCR reaction mixture contained Ultrapure™ DNase-free Distilled Water (Invitrogen, Carlsbad, CA, USA), 1 x PCR buffer (Sigma-Aldrich, St Louis, MO, USA), 1.5 µM MgCl_2_ (Sigma-Aldrich), 0.2 µM of each dNTP (Sigma-Aldrich), 2.5 U Taq DNA Polymerase (Sigma-Aldrich), 2.5 µL of BSA (0.1 g/10 mL), 1 pmol of each forward and reverse primer, and 5 µL of extracted DNA in a total reaction volume of 50 µL. After denaturation at 94ºC for 3 min, the first PCR consisted of 35 cycles (denaturation at 94ºC for 30 s, annealing at 57ºC for 30 s, and extension at 72ºC for 40 s), followed by a final extension at 72ºC for 10 min. Conditions for the second PCR were identical to the first PCR, except for the following modifications: 30 cycles with an annealing temperature of 55ºC. Also, 5 µL of amplified DNA produced from the first PCR was used as template.

Negative and positive controls were included in all PCR reactions. Positive control samples were obtained from faecal samples from immunocompromised patients positive for *E. bieneusi* from the Clinical Hospital. PCR products were subjected to electrophoresis in agarose gel (2%) with the Low DNA Mass Ladder (Invitrogen), and visualised under UV light (UVDI-365®, Major Science) after staining the gel with GelRed^®^ (Biotium, Fremont, CA, USA).

All positive PCR products were purified using the ExoSAP-IT (USB Corporation, Cleveland, OH, USA), following the manufacturer’s instructions. Both strands were sequenced using the inner primers utilising the Applied Biosystems® 3500 Genetic Analyzer (Helixxa, São Paulo, Brazil).

Sequences obtained were aligned with reference sequences downloaded from GenBank using the program MEGA version 7 (Kumar et al. 2016) to determine *E. bieneusi* genotypes. Phylogenetic and molecular evolutionary analyses were made using MEGA version 7 (Kumar et al. 2016). Phylogenetic inference was determined by the neighbor-joining (NJ) method of Saitou and Nei (1987). Genetic distance was calculated with the Kimura 2-parameter model. The GenBank accession numbers of the reference sequences are: AF 101200, AY371286, AF348475, AY371283, AF242478, AY371281, AF076042, AY237223, AF101199, AF076040, AF076043, AY331007, EU153584, FJ439683, KY363360, JN997481, JN595887, JN997479, JN997480, DQ683749, JQ437575.

## RESULTS

**Fig. 4:**
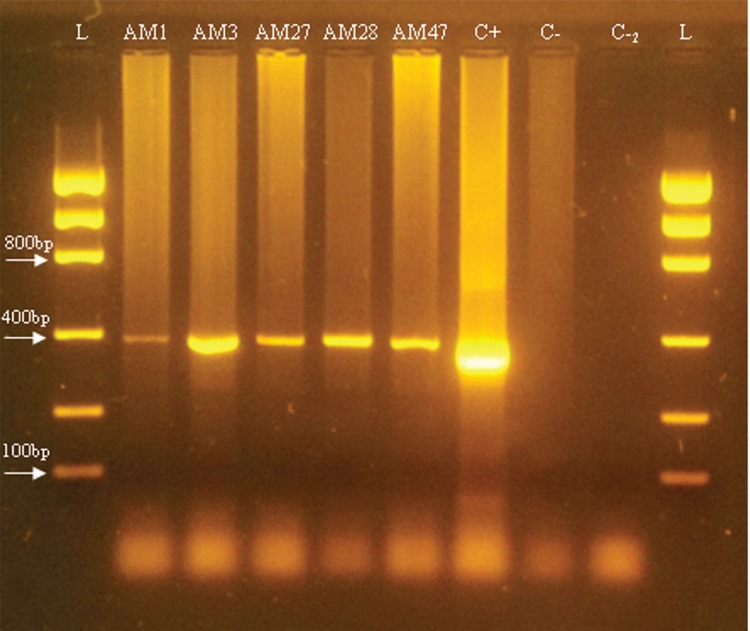
: agarose gel electrophoresis (2%) showing amplification products from nested polymerase chain reaction (PCR) of *Enterocytozoon bieneusi*. L: Low DNA Mass Ladder; arrows indicate fragments of 100 bp, 400 bp and 800 bp; AM1; AM3 and AM27: raw sewage; AM28 and AM 47: treated effluent; C+: positive control; C-: first negative control; C-2: second negative control. Size of the fragment amplified from *E. bieneusi* 18S rRNA: 390 bp.

From the 18 raw sewage samples analysed by nested PCR (Table), three (16.6%) were positive for *E. bieneusi* (AM1, AM3, AM27), whereas out of 18 treated effluent samples (Table), two (11.1%) were positive (AM28, AM47) (Fig. 4).

**TABLE:** Nested polymerase chain reaction (PCR) results of *Enterocytozoon bieneusi* and genotype in raw sewage and treated effluent samples from combined system for wastewater treatment

Raw sewage samples	Amplification	Genotype	Treated effluent samples	Amplification	Genotype
AM1	+	Not determined	AM2	-	-
AM3	+	Not determined	AM4	-	-
AM5	-	-	AM6	-	-
AM7	-	-	AM8	-	-
AM9	-	-	AM10	-	-
AM13	-	-	AM11	-	-
AM14	-	-	AM12	-	-
AM15	-	-	AM17	-	-
AM20	-	-	AM18	-	-
AM21	-	-	AM19	-	-
AM27	+	EbRB	**AM28**	+	Not determined
AM33	-	-	AM22	-	-
AM34	-	-	AM23	-	-
AM35	-	-	AM24	-	-
AM36	-	-	AM26	**-**	-
AM37	-	-	AM32	-	-
AM38	-	-	AM47	+	EbRB
AM46	-	-	AM48	-	-

+: positive; -: negative; samples in bold: AM28 is correspondent to AM27.

**Fig. 5:**
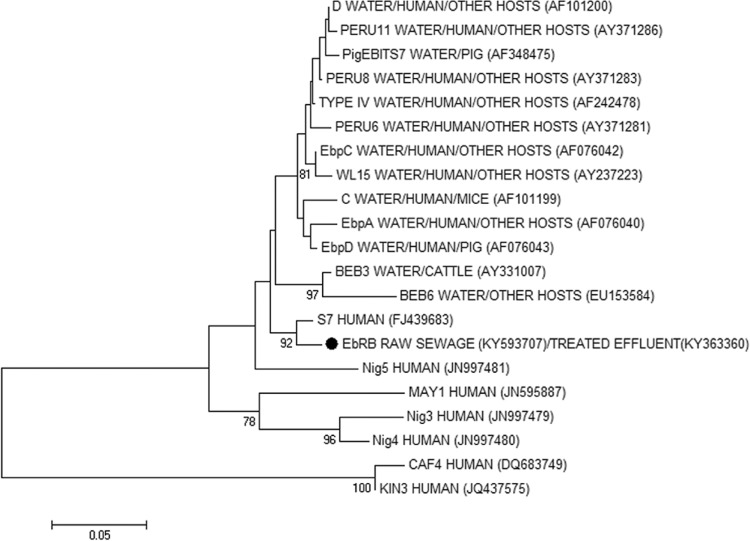
: phylogenetic relationships among the novel *Enterocytozoon bieneusi* genotype (EbRB, identified with a ●) found in raw sewage and treated effluent, and *E. bieneusi* genotypes previously identified in water, humans and/or other hosts as inferred by a neighbor-joining analysis of the internal transcriber spacer (ITS) rRNA gene sequence, based on genetic distances calculated by the Kimura two-parameter model. Bootstrap values of less than 75% are not shown.

After sequencing all five positive samples, the *E. bieneusi* sequence was verified in four samples (AM3, AM27, AM28, and AM47). Sequencing of samples AM27 and AM47 revealed a novel *E. bieneusi* genotype (EbRB). The closest match for EbRB is genotype S7 (FJ439683) (syn. CHY1 KT267289) (Fig. 5), isolated from humans in the Netherlands (ten Hove et al. 2009), with five nucleotide differences. These sequences were deposited in GenBank under Accession Numbers KY363360 and KY593707. AM1, AM3, and AM28 were not successfully sequenced; hence, it was not possible to determine their genotypes.

## DISCUSSION

In Brazil, prevalence surveys of HIV+ patients with diarrhoea showed that 5%, 27.5%, and 46.1% were infected by microsporidia (Wuhib et al. 1994, Brasil et al. 1996, 2000). Microsporidian spores were also identified in 1.3% of immunosuppressed patients in the city of Goiânia (Goiás, Brazil) (de Souza Jr & García-Zapata 2006), and in 36% of rheumatic disease patients undergoing anti-tumour necrosis factor therapy/anti-rheumatic drug treatment as compared to 4% of controls without diarrhoea (Aikawa et al. 2011). In 2013, Costa et al. (2013) described high positivity (40.3%) for microsporidia in patients who underwent transplantation surgery in the University Clinical Hospital, including bone marrow (75%), kidney (22%), and liver (3%) transplantation. These studies have shown that microsporidiosis is associated with chronic diarrhoea in immunocompromised patients and other immunosuppressed individuals in Brazil.

*E. bieneusi* was confirmed by PCR and electron microscopy as the only enteric pathogen found in five of 11 patients included in a cohort study of 40 HIV+ individuals with chronic diarrhoea in Rio de Janeiro state (Brasil et al. 2000). One HIV+ patient exhibited chronic diarrhoea and a CD4 level < 100 cells/mm^3^ (Brasil et al. 1998).

Although molecular studies of microsporidia in Brazil are still incipient, some investigations have demonstrated that *E. bieneusi* is a common species parasitising animals in this country. There are reports in cattle (Fiuza et al. 2016a), pigs (Fiuza et al. 2015), sheep (Fiuza et al. 2016b), coatis (Lallo et al. 2012a), and birds (Lallo et al. 2012b, Cunha et al. 2016). These findings highlight the risk of human microsporidiosis by zoonotic transmission, since genotypes previously reported in both humans and animals were found in all animals surveyed in these studies, with the exception of coatis.

Many epidemiological studies have identified the consumption of untreated water as a potential risk factor for microsporidiosis in humans (Didier et al. 2004, Wumba et al. 2012), and *E. bieneusi* spores have been found in surface water at relatively high frequencies (Graczyk et al. 2010, Galván et al. 2013, Guo et al. 2014). Guo et al. (2014) found that 58.2% of storm water samples in the United States were positive for *E. bieneusi* using filtration and PCR. In China, Hu et al. (2014) found *E. bieneusi* in 31.5% of drinking water samples, after episodes of water contamination with carcasses of domestic pigs. Galván et al. (2013) reported that 61% of domestic effluent samples in Madrid, Spain, contained microsporidia such as *Encephalitozoon intestinalis*, *Encephalitozoon cuniculi*, *Anncaliia algerae*, and *E. bieneusi* (genotypes C and D).

Although preliminary, our findings are relevant since this is the first report of *E. bieneusi* occurrence in environmental samples in Brazil. DNA of microsporidian spores was amplified from two samples (AM27 and AM28), revealing the presence of *E. bieneusi* in both raw sewage and corresponding treated effluent samples. This finding demonstrates that the combined system did not achieve complete removal of *E. bieneusi* spores. This issue can be attributed to the low sedimentation rate of the spores in the decanted liquid of the combined system, thus they remained in the treated effluent.

Until date, over 201 genotypes of *E. bieneusi* have been reported based on nucleotide polymorphisms in the ITS region of the rRNA gene (Fayer & Santín-Duran 2014). While some genotypes are host-specific for humans, others show host specificity for animals, and some are zoonotic (Santín & Fayer 2011).

A novel *E. bieneusi* genotype (EbRB) was identified in a raw sewage (AM27) and a treated effluent (AM47) sample. The risks of human infection for this genotype are unknown. The closest match for this new strain is genotype S7, which was previously reported in a diabetic patient exhibiting severe diarrhoea and rectal bleeding (ten Hove et al. 2009), in horses (Santín et al. 2010), and in a yak (*Bos grunniens*) in a zoo in China (Li et al. 2015). Henriques-Gil et al. (2010) suggested that some genotypes of *E. bieneusi* circulate within a specific host species and are only occasionally transmitted to another host.

Furthermore, contaminated water is a possible source of infection for transplant and HIV+ patients. Sewage discharges are a significant risk factor for the introduction of human enteropathogens into surface waters (Graczyk & Lucy 2007), and microsporidia spores are environmentally robust and ubiquitous in aquatic habitats (Graczyk et al. 2007). In addition, the present finding of *E. bieneusi* in treated effluent from the University Clinical Hospital is important from both public health and environmental standpoints because this final effluent flows into the Ribeirão das Pedras stream, which runs through several neighbourhoods, and is discharged into the Anhumas stream, a tributary of the Atibaia River, the main water supply of the city of Campinas.

By using *E. intestinalis* in their assays, Gerba et al. (2003) evaluated spore removal through a conventional water treatment process on a pilot scale, and reported a high removal efficiency (2.47 log) using coagulation and sedimentation steps followed by a mixed media filtration. However, spore size can influence the removal efficiency attained by water and wastewater treatment plants, and *E. bieneusi* spores are smaller than those of *E. intestinalis*.

Negative removal efficiencies were obtained for *E. bieneusi* in two plants (-100%) by Cheng et al. (2011), who also reported high concentrations of microsporidian spores in inflowing wastewaters and in wastewater processing end-products (biosolids and final effluents) in Ireland. Therefore, spore identification, removal, and inactivation in drinking water and wastewater treatment processes remain technological challenges (Graczyk & Lucy 2007).

Microsporidia were included in the United States Environmental Protection Agency’s Contaminant Candidate List in 1998 and 2005 (Guillot & Loret 2009), and are currently considered as Category B priority pathogens by the National Institute of Allergy and Infectious Diseases (NIAID, National Institutes of Health, Bethesda, MD, USA - niaid.nih.gov/topics/biodefenserelated/biodefense/pages/cata.aspx) due to their impact on public health. However, the Brazilian Drinking Water Legislation (MS 2011) (Federal Decree 2914/Health Ministry) published in 2011 did not address microsporidia monitoring in the water supply.

The presence of *E. bieneusi* spores in the final effluent reported in this study suggests that *E. bieneusi* contamination by human sources may be occurring at the Atibaia River basin. Thus, enteropathogen source-tracking research in wastewater treatment plants is critical, considering that surface water supplies in Brazil receive large amounts of raw sewage (Sodré et al. 2010).
